# Case Report: Seizure freedom after resection of a temporal choroidal fissure cyst in drug-resistant epilepsy

**DOI:** 10.3389/fnins.2026.1905779

**Published:** 2026-09-08

**Authors:** Guanglin Fu, Fan Ye, Shanshan Lu, Xingwang Sun, Peng Zhou

**Affiliations:** 1 Department of Neurosurgery, The First Affiliated Hospital of Shandong First Medical University & Shandong Provincial Qianfoshan Hospital, Jinan, China; 2 Shandong First Medical University, Jinan, China; 3 Department of Neurology, The First Affiliated Hospital of Shandong First Medical University & Shandong Provincial Qianfoshan Hospital, Shandong Institute of Neuroimmunology, Jinan, China; 4 Department of Pain, Shandong Cancer Hospital and Institute, Shandong First Medical University & Shandong Academy of Medical Sciences, Jinan, China

**Keywords:** choroidal fissure cyst, drug-resistant epilepsy, SEEG, surgical resection, temporal lobe epilepsy

## Abstract

**Background:**

Choroidal fissure cysts (CFCs) are relatively rare, and their exact prevalence in the general population remains unclear. These cysts are most often detected incidentally during neuroimaging performed for unrelated reasons. Whether CFCs are associated with specific clinical symptoms and their potential clinical significance remain controversial.

**Case description:**

A 35-year-old woman developed epileptic seizures at age 15 after a high fever. Her seizures remained poorly controlled despite multiple antiseizure drugs. Neuroimaging revealed a choroidal fissure cyst in the right hippocampal region. Stereoelectroencephalography (SEEG) confirmed epileptogenic foci adjacent to the cyst, and SEEG-guided radiofrequency thermocoagulation (RF-TC) was performed initially. She remained seizure-free after RF-TC until definitive surgery, when anterior temporal lobectomy was performed. Available tissue specimens showed no definite histopathological evidence of ILAE-classifiable focal cortical dysplasia or hippocampal sclerosis. At the 12-month postoperative follow-up, she remained seizure-free; outpatient video-electroencephalography showed no significant abnormalities, and emotional status and cognitive function had markedly improved from the preoperative baseline. Postoperatively, she continued lacosamide 150 mg twice daily and lamotrigine 100 mg twice daily. The outcome was Engel Class IA and ILAE Class 1.

**Conclusion:**

We identified a rare epileptogenic origin. Neuroimaging revealed a right-sided choroidal fissure cyst corresponding to the epileptogenic zone. Combined SEEG and intraoperative ECoG, used for the first time, confirmed an epileptogenic focus adjacent to the cyst, with seizures originating from the hippocampal head and body. The available histopathological specimens showed no definite evidence of hippocampal sclerosis or ILAE-classifiable focal cortical dysplasia (FCD). These findings suggest that, in certain cases, choroidal fissure cysts may act as independent epileptogenic triggers, with implications for epilepsy diagnosis and management.

## Introduction

CFCs are rare congenital developmental anomalies that can occur at any age but are more frequently observed in adults. They are located within the choroidal fissure between the fornix and thalamus (Yamila et al., 2023). During embryonic development, residual choroid plexus structures may undergo cystic expansion, resulting in CFCs (Altafulla et al., 2020). Previous authors have reported a possible association between CFCs and epileptic seizures (Morioka et al., 1994, 1998); however, none underwent invasive SEEG verification or surgical intervention, and causality could not be established. We report a case of drug-resistant epilepsy with a CFC in the temporal horn ipsilateral to the epileptogenic focus. Dual functional validation combining spontaneous seizure recordings on SEEG and electrical stimulation–induced responses, together with RF-TC therapeutic validation and long-term seizure freedom after definitive surgical resection, established the most complete chain of localization evidence to date in CFC-associated epilepsy. These findings suggest that the drug-resistant epilepsy may be associated with the CFC.

### Case demonstration

The patient was a 35-year-old right-handed woman born via full-term spontaneous vaginal delivery without perinatal hypoxia. Her past medical and family histories were unremarkable, with no family history of epilepsy or other neurological disorders. Her first seizure occurred 20 years earlier, at age 15, after a high fever. It manifested as bilateral hand clenching with leftward torso rotation and lasted approximately 1 min. She was amnestic for the event and had no significant postictal discomfort. Because the symptoms were mild, she did not seek medical attention or treatment.

She subsequently developed nocturnal seizures characterized by sudden sitting up during sleep, hand fumbling with vocalization, impaired awareness, unresponsiveness to verbal stimuli, and inability to communicate. The episodes resolved spontaneously after several tens of seconds. Oral carbamazepine provided partial relief, but seizures persisted irregularly.

Seizure frequency gradually increased to approximately 2–3 episodes per week. Occasional daytime seizures involved sudden falls, loss of consciousness, and progression to bilateral tonic–clonic seizure, followed by sitting up, bilateral manual automatisms (hand fumbling), and screaming. Episodes lasted around 2 min. Postictal headache and fatigue resolved after rest. Carbamazepine 400 mg/day was replaced with oxcarbazepine 300 mg twice daily plus lamotrigine 50 mg twice daily, but seizure control remained poor.

After admission to our hospital, despite treatment with multiple antiseizure medications—sodium valproate (250 mg in the morning and 500 mg at night), lamotrigine (150 mg twice daily), clonazepam (1 mg twice daily), lacosamide (100 mg twice daily), and zonisamide (100 mg at night)—the patient’s seizures remained poorly controlled.

Physical and neurological examinations and laboratory tests on admission were normal. HAMA, HAMD-24, and MoCA scores were 22, 32, and 21, indicating marked anxiety, moderate depression, and mild cognitive impairment, respectively; the cognitive impairment may have been related to chronic epilepsy and antiseizure polytherapy. Video-EEG showed occasional interictal spike-and-sharp-wave discharges over the right temporal region during wakefulness and sleep, maximal at F8, T2, and T4. Eight seizures of two types were recorded. Type I seizures involved leftward head deviation after arousal, followed by bilateral upper-limb tonic stiffening, vocalization, and impaired awareness, lasting 10–20 s. Ictal EEG showed evolving 5–6 Hz rhythmic sharp waves over the right temporal region, preceding clinical onset by about 15–16 s. Type II seizures involved bilateral hand fumbling or automatisms, upper-limb shaking, vocalization, and impaired awareness, lasting 3–20 s without reliable EEG lateralization. Type I seizures were considered right temporal in origin, whereas Type II seizures remained nonlateralized.

**Figure 1:**
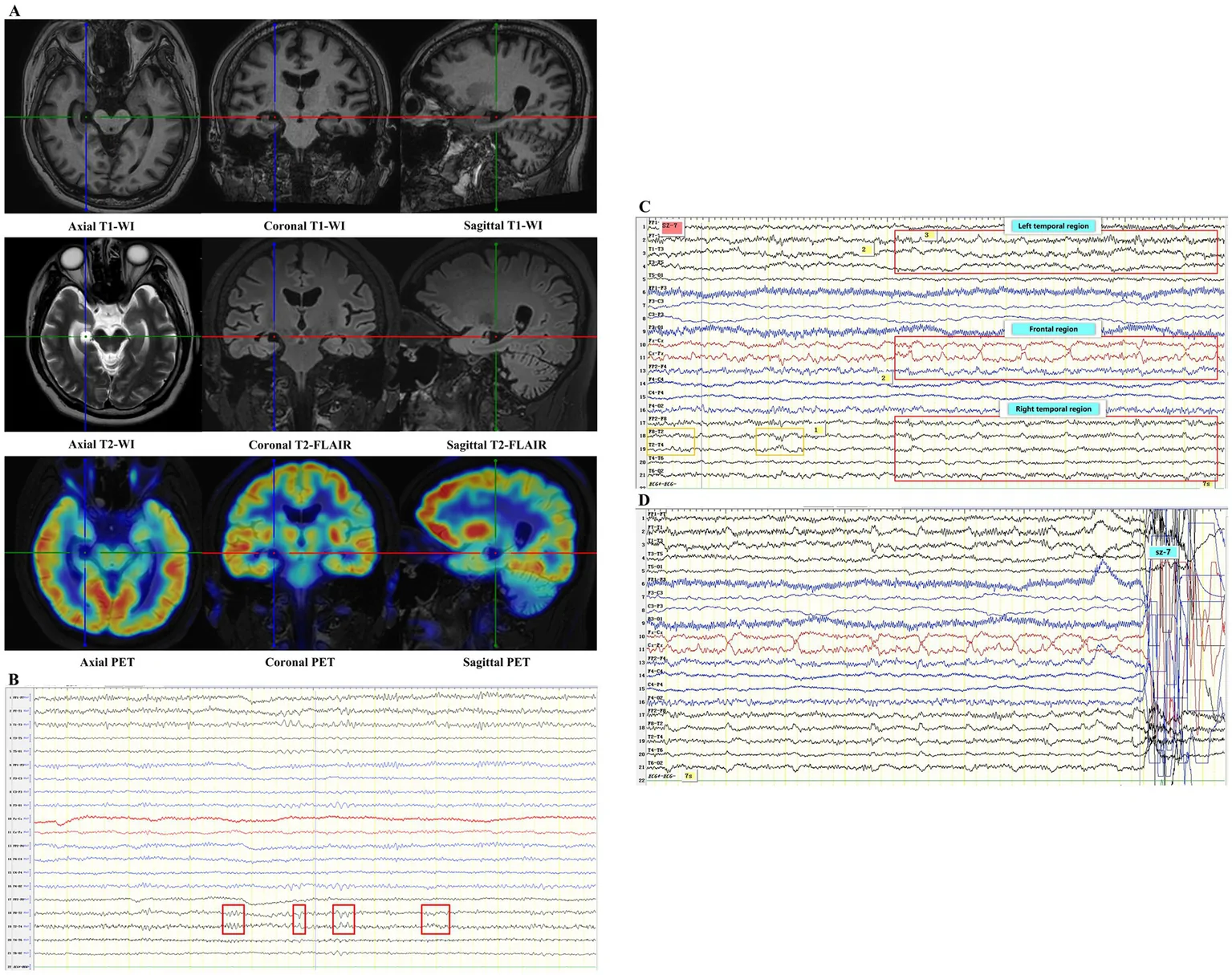
Preoperative imaging and scalp video-EEG findings. **(A)** Axial, coronal, and sagittal structural MRI and co-registered PET images demonstrate a right choroidal fissure cyst adjacent to the medial aspect of the hippocampus, with mild displacement of the hippocampus and hypometabolism in the ipsilateral hippocampal-amygdalar region. The intersection of the linked crosshairs indicates the center of the cyst identified on MRI and the corresponding anatomical coordinates on the co-registered PET images. **(B)** Interictal EEG showed sharp-wave discharges over the right anterior temporal region, maximal at T2, with phase reversal between the F8-T2 and T2-T4 derivations. **(C)** Approximately 15 s before clinical onset, rhythmic small sharp waves emerged over the right temporal region, rapidly propagated to the right frontal and left anterior temporal regions, and subsequently involved both temporal regions. **(D)** During the clinical seizure, rhythmic sharp-wave activity was observed over the bilateral temporal regions, followed by prominent movement, electromyographic, and electrode artifacts.

Brain MRI showed a 1.2 × 1.2 cm cystic lesion in the medial right temporal lobe near the hippocampus. It was hypointense on T1WI, showed cerebrospinal fluid–like hyperintensity on T2WI, and was almost completely suppressed on T2-FLAIR, without wall thickening, enhancement, surrounding edema, or other aggressive features. Multiplanar images showed that the lesion was outside the hippocampal formation and mildly displaced the adjacent hippocampus, favoring a choroidal fissure cyst over a hippocampal sulcus remnant cyst. The right hippocampus showed mild volume loss and slightly increased T2WI signal but lacked the segmental atrophy and marked T2/FLAIR hyperintensity typical of hippocampal sclerosis. PET/MRI showed hypometabolism in the right amygdala and hippocampus adjacent to the cyst (Figure 1).

**Figure 2:**
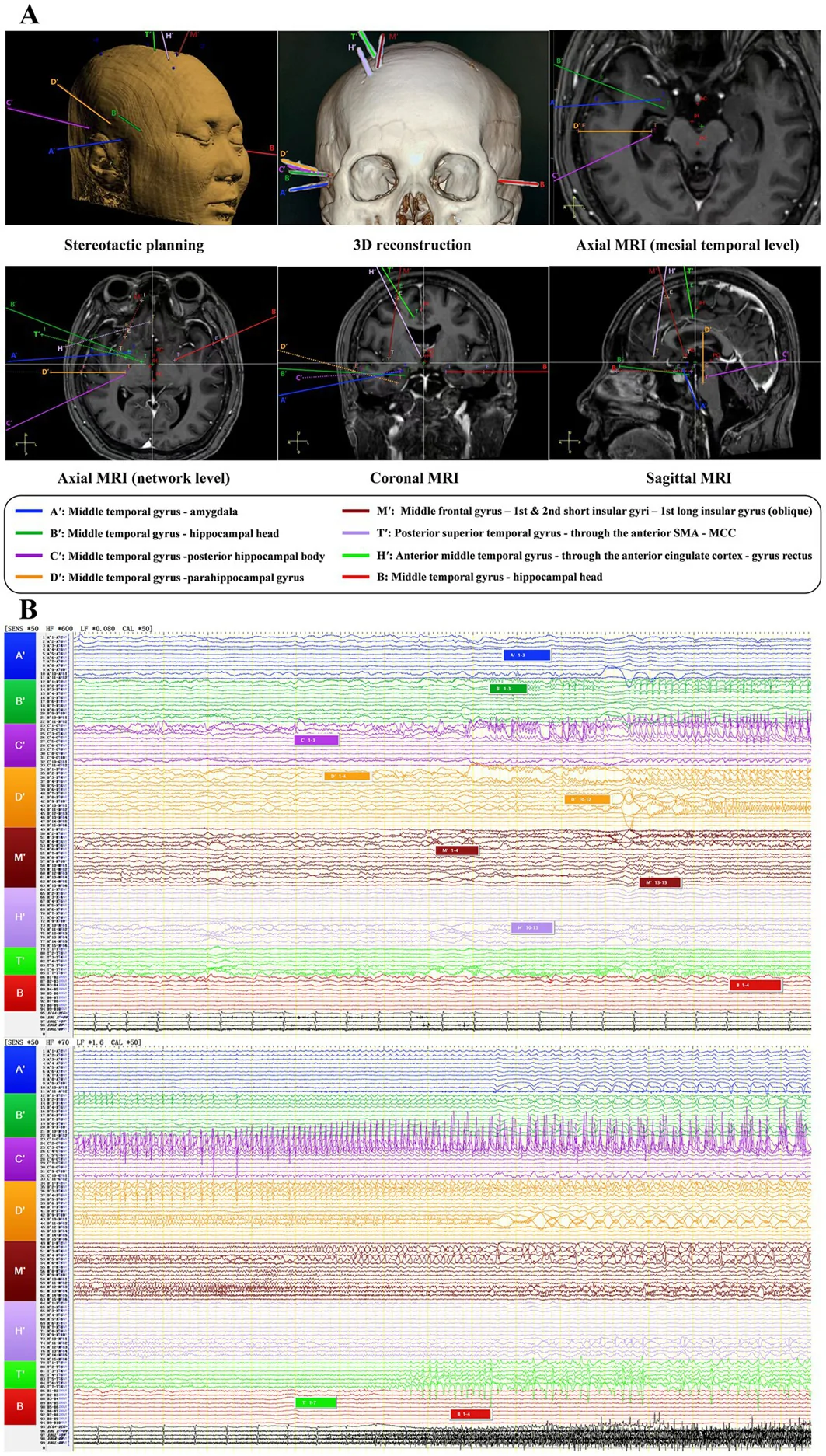
SEEG electrode implantation and representative ictal recordings. **(A)** Three-dimensional reconstruction and multiplanar MRI showing the implantation configuration and intracranial trajectories of the SEEG electrodes. **(B)** Postoperative SEEG recordings demonstrated that the right hippocampus and parahippocampal gyrus were the seizure onset zones. The onset of abnormal epileptiform discharges recorded by the right deep electrodes corresponded to the lesion identified on MRI.

To localize the seizure onset zone, SEEG was performed with seven electrodes implanted in the right frontotemporal region and one in the left temporal region. Fifteen seizures were recorded after implantation (Figure 2). Twelve originated from contacts C′1–4, B′1–3, or D′1–4, and three from the middle temporal gyrus, representing two distinct ictal onset patterns. The first pattern occurred during sleep and was characterized by leftward head turning, proximal rigidity of both upper limbs, distal tremor with vocalization, and progression to a bilateral tonic–clonic seizure lasting approximately 1–2 min. The earliest electrographic change was 7–14 Hz rhythmic sharp-wave activity in contacts C′1–4 within the posterior body of the right hippocampus, with early recruitment of deep insular contacts M′1–4 and superficial middle frontal gyrus contacts M′11–15. Subsequently, 40–50 Hz low-amplitude fast activity appeared in M′11–15, whereas C′1–4 evolved into an approximately 8 Hz continuous sharp-wave rhythm. Discharges then sequentially recruited hippocampal head contacts B′1–4, parahippocampal gyrus contacts D′1–4, and the supplementary motor area–midcingulate pathway (T′1–8), before propagating to the contralateral hippocampus and widespread bilateral networks. Electrographic onset preceded clinical onset by approximately 15–17 s. The second pattern occurred during wakefulness and was characterized by leftward head turning, asymmetric tonic posturing with limb shaking and fear-like vocalization, followed by bilateral manual automatisms; some seizures progressed to bilateral tonic–clonic activity. The earliest electrographic change was ~35 Hz fast activity in superficial right middle temporal gyrus contacts D′10–12, A′10–11, and B′10–11. This was followed by recruitment of M′11–15 and M′1–4, with 14–16 Hz rhythmic sharp-wave activity and 40–50 Hz low-amplitude fast activity. Discharges rapidly spread to posterior hippocampal body contacts C′1–4, hippocampal head contacts B′1–4, parahippocampal gyrus contacts D′1–4, and T′1–8, followed by widespread bilateral network involvement. Clinical manifestations appeared approximately 30 s after electrographic onset.

To further verify the epileptogenic zone and define eloquent cortex boundaries, electrical stimulation was performed through implanted electrodes. Bipolar stimulation was applied (50 Hz, 0.5 ms pulse width, 3–5 s duration; current gradually increased from 0.5 to 3 mA). Stimulation of the right hippocampal head (B′1–2) and body (C′1–2) induced the habitual aura (leftward head-turning sensation and epigastric discomfort), consistent with spontaneous seizure onset. Stimulation of the right amygdala (A′1–3) elicited a similar but milder aura, suggesting an early propagation zone. Stimulation of the superficial right middle frontal gyrus (M′12–15) and contralateral hippocampus (B) produced no symptoms. Based on spontaneous seizures and stimulation results, the epileptogenic network involved the right hippocampal head (B′1–3), body (C′1–4), amygdala (A′1–3), and parahippocampal gyrus (D′1–4), with possible involvement of the right insular and superficial middle frontal gyrus contacts (M′5–6 and M′11–15). Stereotactic RF-TC was performed on these contacts (two sessions, 5 W, 30 s). The patient remained seizure-free during the one-month interval before anterior temporal lobectomy, further supporting SEEG localization and confirming these targets as critical nodes of the epileptogenic network.

**Figure 3:**
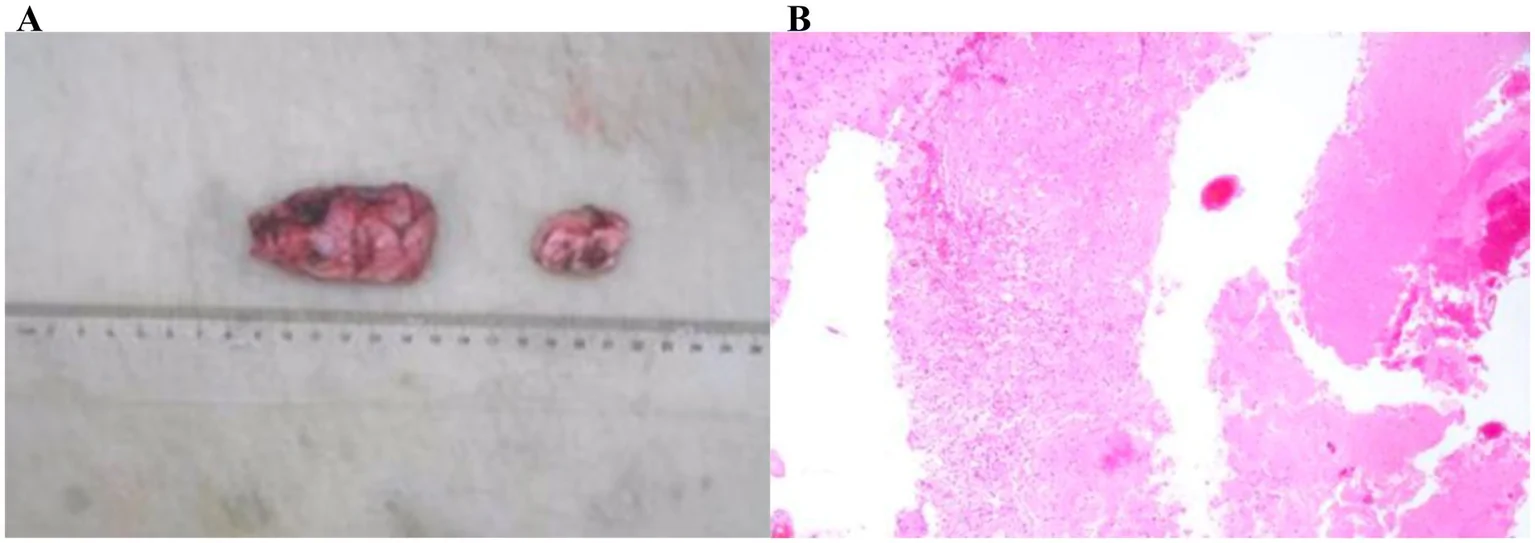
Gross pathological specimen and hematoxylin–eosin (HE) staining (×200). **(A,B)** Multifocal infarcts with hemorrhage were observed in the cerebral cortex and subcortical white matter (temporal pole, hippocampus, and amygdala), as well as in portions of hippocampal structures, accompanied by abundant macrophage and lymphocyte infiltration, along with gliosis and small vessel proliferation, consistent with changes following electrode implantation. The hippocampal tissue was sparse, with focal areas showing mild neuronal loss accompanied by gliosis and small vessel proliferation.

Following multidisciplinary discussion, SEEG showed that the seizure-onset and early propagation network involved the right hippocampal head and body, amygdala, parahippocampal gyrus, and several superficial lateral temporal contacts. Given the possible persistent compression and irritation caused by the choroidal fissure cyst and the potentially limited long-term seizure control provided by RF-TC alone, the patient underwent right anterior temporal lobectomy with resection of the mesial temporal structures and cyst 1 month after RF-TC to address both the epileptogenic network and structural lesion. Intraoperative ECoG guided the extent of resection. The cyst in the right hippocampal region was removed together with the right hippocampal head and body, and the specimens were submitted for histopathological examination (Figure 3). The patient recovered well without new neurological deficits.

Neuropsychological assessment performed 10 days after surgery showed the following: the HAMA score decreased to 14, an 8-point reduction from the preoperative score of 22 (marked anxiety), suggesting notable relief of anxiety symptoms without full remission; the HAMD-24 score dropped to 11 (mild depression), a 21-point decline from the preoperative score of 32 (moderate depression), indicating substantial improvement in depressive symptoms; the MMSE score was 28 (with ≥27 considered normal), whereas the MoCA score remained at 21 (mild cognitive impairment, score range 25–21); and the Activities of Daily Living (ADL) scale score was 23.

At the 12-month postoperative follow-up, the patient remained on oral lacosamide (150 mg twice daily) and lamotrigine (100 mg twice daily) and remained seizure-free; outpatient video-EEG revealed no significant abnormalities, with seizure outcome classified as Engel Class IA and ILAE Class 1. Follow-up neuropsychological assessment showed that the HAMA score decreased to 11, the HAMD-24 score dropped to 9 (with a score of <8 indicating no depression), and the MoCA score improved to 23. The ADL score increased to 24. Although anxiety and depressive symptoms had markedly improved from the preoperative baseline, they had not fully normalized, suggesting that psychological impairment resulting from prolonged seizure burden may require a longer recovery trajectory. Cognitive function gradually improved but remained within the mild impairment range on the MoCA (21–25), which may be attributable to the concurrent use of multiple antiseizure medications and the residual cognitive impact of long-standing preoperative epileptic activity.

## Discussion

CFCs are benign intracranial cystic lesions that occur at the level of the choroidal fissure (Altafulla et al., 2020). The choroidal fissure, along which the choroid plexus of the temporal horn is attached, is located at the junction of the roof and lateral wall of the middle incisural space, bounded inferiorly by the fimbria and superiorly by the pulvinar, contains cerebrospinal fluid, and follows an oblique course from posterosuperior to anteroinferior (Holodny et al., 1998; Tubbs et al., 2012). During fetal development, abnormalities in the formation or migration of the choroid plexus may lead to the development of choroidal fissure cysts.

Currently, MRI is the preferred modality for identifying and localizing choroidal fissure cysts, which typically demonstrate signal characteristics similar to cerebrospinal fluid, without contrast enhancement, surrounding edema, or gliosis (Karatas et al., 2016). On axial images, these cysts usually appear as intra-axial cystic lesions; their relationship with the choroidal fissure is better delineated on coronal images, while they often exhibit a spindle-shaped configuration on sagittal images Therefore, they should be differentiated on imaging from other lesions, such as cerebral infarction and hippocampal sulcus remnant cysts. Most cysts are small and asymptomatic, often discovered incidentally; however, in rare cases, they may compress the hippocampus and cause focal ischemia, thereby potentially serving as an epileptogenic focus (Lang et al., 2024).

Previous studies have suggested a potential association between choroidal fissure cysts and epilepsy. Sherman et al. reported that 5 out of 26 patients with choroidal fissure cysts had seizures; however, the authors concluded that no definite association could be established between the cysts and epilepsy (Sherman et al., 1990). However, Achour et al. (2020), Isolan et al. (2010), and Morioka et al. (1994) reported a possible association between choroidal fissure cysts and epileptic seizures. Ventura et al. reported two cases of choroidal fissure cysts associated with myoclonic seizures (Ventura et al., 2011). In these cases, the cysts were all located in the right choroidal fissure, and ictal electroencephalography demonstrated generalized spike-and-wave discharges, suggesting a possible relationship between the cysts and seizures, however, none of the cases underwent surgical intervention or invasive SEEG verification, and therefore a definite causal relationship could not be established (Al-Holou et al., 2013) (Table 1).

**Table 1:** Summary of patients with choroidal fissure cyst combined with epilepsy in the literature.

Author (year)	Age	Gender	Age of onset (years)	Clinical manifestation	Size of the CFC (cm)	Site of the CFC	EEG	Management
Achour et al. (2020)	8	F	5	Automatism (chewing)	NR	R	Normal	Carbamazepine
Isolan et al. (2010)	18	M	18	Oral automatism confusion with amnesia	NR	L	Normal	Carbamazepine 400 mg/day
Isolan et al. (2010)	21	F	7	Loss of consciousness oral and manual automatisms eneralized tonicclonic seizures	NR	L	Normal	Carbamazepine 1,200 mg/day Phenobarbital 100 mg/day
Arroyo and Santamaria (1997)	24	NR	15	NR	1 × 0.6 × 0.5	R	Five seizures: right parietal lobe onset	Conservative treatment
Arroyo and Santamaria (1997)	35	NR	32	NR	0.5 × 0.5 × 0.5	R	Not performed	Conservative treatment
Arroyo and Santamaria (1997)	39	NR	35	NR	0.3 × 0.3 × 0.3	R	Not performed	Conservative treatment
Arroyo and Santamaria (1997)	22	NR	2	NR	0.5 × 0.5 × 0.5	R	Four seizures: frontal lobe onset (not definite side of preference)	Conservative treatment
Arroyo and Santamaria (1997)	10	NR	9	NR	1.5 × 1.5 × 0.15	R	Not performed	Conservative treatment
Arroyo and Santamaria (1997)	24	NR	Neonate	NR	0.5 × 0.5 × 0.5	L	Two seizures: right fronto-central onset	Conservative treatment
Arroyo and Santamaria (1997)	46	NR	31	NR	1.5 × 1 × 1	R	Not performed	Conservative treatment
Arroyo and Santamaria (1997)	46	NR	8	NR	1 × 0.5 × 0.5	L	Not performed	Conservative treatment
Arroyo and Santamaria (1997)	51	NR	14	NR	5 × 3.5 × 2.5	R	Not performed	Conservative treatment
Arroyo and Santamaria (1997)	40	NR	29	NR	2.5 × 3 × 2.5	R	Two seizures: right frontal lobe onset	Conservative treatment
Arroyo and Santamaria (1997)	18	NR	4	NR	2 × 2 × 2	L	Not performed	Conservative treatment
Arroyo and Santamaria (1997)	26	NR	13	NR	0.5 × 0.5 × 0.5	R	Two seizures: left frontal lobe onset	Conservative treatment
Arroyo and Santamaria (1997)	24	NR	7 months	NR	0.5 × 0.5 × 0.5	R	Two seizures: diffuse onset (bilateral fronto-temporal)	Resect
Morioka et al. (1994)	22	M	16	Disturbance of ideatation	NR	R	Sporadic sharp waves on right temporal region with intermittent delta and theta activities on the bifrontal area	Phenytoin Valproate
Morioka et al. (1994)	28	F	10	Olfactory hallucinations, consciousness with automatic behavior and amnesia	NR	L	Occasional sharp waves on the left fronto-temporal region	Carbamazepine Valproate

M, male; F, female; NR, no report; R, right; L, left.

The patient’s clinical presentation was consistent with temporal lobe epilepsy, and ictal EEG showed paroxysmal discharges in the right temporal region. The novelty of this case lies in the following: for the first time, through prolonged video-EEG monitoring and SEEG evaluation, the precise spatial correspondence between seizure onset and the perilesional region of the cyst was established; seizure freedom after RF-TC and subsequent long-term seizure freedom after anterior temporal lobectomy provide the most substantial evidence to date for an association between the choroidal fissure cyst (CFC) and epilepsy. PET revealed hypometabolism in the right hippocampus, parahippocampal gyrus, and amygdala, consistent with the classic imaging features of hippocampal sclerosis. However, histopathological examination revealed only a reduced amount of hippocampal tissue, focal mild neuronal loss, and proliferation of glial cells and small vessels. The available histopathological findings did not support FCD types I–III, consistent with “no definite FCD on histopathology” (Blümcke et al., 2011; Najm et al., 2022; Blümcke et al., 2013). We propose the following hypothesis: the long-standing mass effect of the CFC leads to chronic neuronal loss, reactive gliosis, and metabolic decline in the compressed hippocampus, which “mimics” hippocampal sclerosis on imaging, yet is pathologically distinct—representing focal, external compressive nonspecific changes rather than diffuse segmental neurodegeneration. The RF-TC lesion is confined to a few millimeters around the electrode contact and is therefore insufficient to erase the typical pathological hallmarks of diffuse hippocampal sclerosis. The available pathological findings did not support a diagnosis of typical hippocampal sclerosis, suggesting that chronic compression by the CFC may be the fundamental cause of the hippocampal imaging abnormalities and epileptogenicity.

The mechanism by which choroidal fissure cysts cause epileptic seizures remains unclear. These cysts may induce epilepsy by compressing adjacent brain tissue, irritating the surrounding cortex, or disrupting cerebrospinal fluid circulation, thereby triggering abnormal neuronal discharges; however, these mechanisms require validation. Mechanical compression may activate hippocampal PIEZO1, which is upregulated in animal models of mesial temporal lobe epilepsy and positively correlated with proinflammatory cytokine expression, suggesting a neuroinflammatory cascade that lowers the neuronal excitability threshold (Garcia et al., 2023). Chronic compression may also disproportionately affect inhibitory interneurons and pyramidal neurons, disrupting the excitation–inhibition balance. Reactive gliosis may impair glutamate homeostasis and enhance local neuronal hyperexcitability (Eid et al., 2004). The “metabolic–electrophysiological dissociation” between hippocampal hypometabolism on PET and hyperexcitability demonstrated by SEEG supports the hypothesis that chronic compression primarily induces functional alterations rather than irreversible structural damage.

At 12 months after surgery, the patient remained seizure-free (Engel Class IA/ILAE Class 1) with no postoperative EEG abnormalities, supporting consideration of antiseizure medication reduction or withdrawal. Previous studies suggest that patients who remain seizure-free after anterior temporal lobectomy may gradually taper and discontinue antiseizure medications, although early complete withdrawal may increase the short-term risk of seizure recurrence (Rathore et al., 2011). Given her 20-year history of drug-resistant epilepsy, preoperative use of more than five antiseizure medications, multiple seizure-onset patterns on SEEG, and short follow-up, continuation of dual therapy remains a cautious, staged strategy. If seizure freedom and stable serial EEG findings persist, one medication may be gradually tapered at a time under specialist supervision and shared decision-making, with an individualized sequence and rate.

Previous imaging studies suggest that choroidal fissure cysts may be more prevalent in patients with epilepsy than in the general population, and seizure reduction or control after surgical resection supports a possible association. This study has limitations. As a retrospective single-case report, causal inference is limited. SEEG has spatial sampling limitations and did not capture all seizure types documented by preoperative video-EEG; thus, the epileptogenic network may not have been fully delineated. Genetic and tissue-based molecular analyses were not performed, precluding assessment of genetic susceptibility and mechanisms involving mechanosensation, inflammation, and glial cells. Original post-stimulation SEEG recordings were unavailable; therefore, post-stimulation discharges and their propagation patterns could not be retrospectively evaluated, limiting the localizing value of electrical stimulation findings. Longitudinal neuropsychological assessments did not use an identical comprehensive test battery, and follow-up remains short. Future multicenter prospective studies incorporating standardized imaging, serial SEEG network analyses, standardized pathological sampling, systematic neuropsychological assessment, and long-term medication-free follow-up are needed to clarify the mechanisms, optimal treatment strategies, and long-term outcomes of CFC-associated epilepsy.

## Patient perspective

From the patient’s perspective, recurrent and unpredictable seizures, including persistent nocturnal events and increasing frequency despite multiple antiseizure medications, affected her daily life. She reported anxiety, uncertainty, and reduced confidence in social and occupational settings. Following comprehensive evaluation and surgical treatment, she experienced complete seizure freedom during follow-up, with improved sense of safety, quality of life, sleep, and ability to engage in daily activities without fear of sudden attacks. She was satisfied with the treatment outcome and more confident in long-term disease control.

## Conclusion

This case identifies a rare epileptogenic origin. Imaging revealed a CFC in the temporal horn ipsilateral to the epileptogenic zone. Dual functional validation combining spontaneous seizure recordings on SEEG and electrical stimulation-induced responses confirmed that the epileptogenic focus was located in the hippocampal head and body adjacent to the cyst. Chronic compression by the CFC may cause hippocampal imaging changes resembling hippocampal sclerosis despite distinct pathology. Multimodal preoperative evaluation helped identify the true epileptogenic focus. SEEG-guided RF-TC followed by anterior temporal lobectomy achieved favorable seizure control. These findings suggest that, under specific circumstances, a CFC may serve as a potential independent epileptogenic trigger and may offer clinical reference value for similar cases.

## Data Availability

The original contributions presented in the study are included in the article/supplementary material, further inquiries can be directed to the corresponding authors.

## References

[ref1] Achour A. Mnari W. Miladi A. Hmida B. Maatouk M. Golli M. . (2020). Temporal choroidal fissure cyst: a rare cause of temporal lobe epilepsy. Pan Afr. Med. J. 36:120. doi: 10.11604/pamj.2020.36.120.21327 PMC7406448 32821331

[ref2] Al-Holou W. N. Terman S. Kilburg C. Garton H. J. Muraszko K. M. Maher C. O. (2013). Prevalence and natural history of arachnoid cysts in adults. J. Neurosurg. 118, 222–231. doi: 10.3171/2012.10.JNS12548, 23140149

[ref3] Altafulla J. J. Suh S. Bordes S. Prickett J. Iwanaga J. Loukas M. . (2020). Choroidal fissure and choroidal fissure cysts: a comprehensive review. Anat Cell Biol. 53, 121–125. doi: 10.5115/acb.20.040, 32647078 PMC7343566

[ref4] Arroyo S. Santamaria J. (1997). What is the relationship between arachnoid cysts and seizure foci? Epilepsia 38, 1098–1102. doi: 10.1111/j.1528-1157.1997.tb01199.x, 9579956

[ref5] Blümcke I. Thom M. Aronica E. Armstrong D. D. Bartolomei F. Bernasconi A. . (2013). International consensus classification of hippocampal sclerosis in temporal lobe epilepsy: a task force report from the ILAE commission on diagnostic methods. Epilepsia 54, 1315–1329. doi: 10.1111/epi.12220, 23692496

[ref6] Blümcke I. Thom M. Aronica E. Armstrong D. D. Vinters H. V. Palmini A. . (2011). The clinicopathologic spectrum of focal cortical dysplasias: a consensus classification proposed by an ad hoc task force of the ILAE diagnostic methods commission. Epilepsia 52, 158–174. doi: 10.1111/j.1528-1167.2010.02777.x, 21219302 PMC3058866

[ref7] Eid T. Thomas M. J. Spencer D. D. Rundén-Pran E. Lai J. C. Malthankar G. V. . (2004). Loss of glutamine synthetase in the human epileptogenic hippocampus: possible mechanism for raised extracellular glutamate in mesial temporal lobe epilepsy. Lancet 363, 28–37. doi: 10.1016/s0140-6736(03)15166-5, 14723991

[ref8] Garcia V. Blaquiere M. Janvier A. Cresto N. Lana C. Genin A. . (2023). PIEZO1 expression at the glio-vascular unit adjusts to neuroinflammation in seizure conditions. Neurobiol. Dis. 187:106297. doi: 10.1016/j.nbd.2023.106297, 37717661

[ref9] Holodny A. I. George A. E. Golomb J. De Leon M. J. Kalnin A. J. (1998). The perihippocampal fissures: normal anatomy and disease states. Radiographics 18, 653–665. doi: 10.1148/radiographics.18.3.9599389, 9599389

[ref10] Isolan G. R. Bianchin M. M. Torres C. M. Bragatti J. A. Assman J. B. Falcetta F. S. (2010). Temporal choroidal fissure cyst and temporal lobe epilepsy: report of two cases. J. Epilepsy Clin. Neurophysiol. 16, 167–169. doi: 10.1590/S1676-26492010000400009

[ref11] Karatas A. Gelal F. Gurkan G. Feran H. (2016). Growing hemorrhagic choroidal fissure cyst. J. Korean Neurosurg. Soc. 59, 168–171. doi: 10.3340/jkns.2016.59.2.168, 26962426 PMC4783486

[ref12] Lang M. Colby S. Ashby-Padial C. Bapna M. Jaimes C. Rincon S. P. . (2024). An imaging review of the hippocampus and its common pathologies. J. Neuroimaging 34, 5–25. doi: 10.1111/jon.13165, 37872430

[ref13] Morioka T. Nishio S. Ishibashi H. Fukui M. (1998). Relationship between arachnoid cysts and seizure foci. Epilepsia 39, 804–805. doi: 10.1111/j.1528-1157.1998.tb01168.x, 9670911

[ref14] Morioka T. Nishio S. Suzuki S. Fukui M. Nishiyama T. (1994). Choroidal fissure cyst in the temporal horn associated with complex partial seizure. Clin. Neurol. Neurosurg. 96, 164–167. doi: 10.1016/0303-8467(94)90054-x, 7924083

[ref15] Najm I. Lal D. Alonso Vanegas M. Cendes F. Lopes-Cendes I. Palmini A. . (2022). The ILAE consensus classification of focal cortical dysplasia: an update proposed by an ad hoc task force of the ILAE diagnostic methods commission. Epilepsia 63, 1899–1919. doi: 10.1111/epi.17301, 35706131 PMC9545778

[ref16] Rathore C. Panda S. Sarma P. S. Radhakrishnan K. (2011). How safe is it to withdraw antiepileptic drugs following successful surgery for mesial temporal lobe epilepsy? Epilepsia 52, 627–635. doi: 10.1111/j.1528-1167.2010.02890.x, 21219315

[ref17] Sherman J. L. Camponovo E. Citrin C. M. (1990). MR imaging of CSF-like choroidal fissure and parenchymal cysts of the brain. AJR Am. J. Roentgenol. 155, 1069–1075. doi: 10.2214/ajr.155.5.2120937, 2120937

[ref18] Tubbs R. S. Muhleman M. McClugage S. G. Loukas M. Miller J. H. Chern J. J. . (2012). Progressive symptomatic increase in the size of choroidal fissure cysts. J. Neurosurg. Pediatr. 10, 306–309. doi: 10.3171/2012.7.PEDS11515 22900488

[ref19] Ventura N. D'Andrea I. Cardoso M. F. Alves-Leon S. V. Gasparetto E. L. (2011). Arachnoid cysts and absence epilepsy: an evidence or a coincidence? Arq. Neuropsiquiatr. 69, 262–263. doi: 10.1590/s0004-282x2011000200024, 21537573

[ref20] Yamila B. M. Juan Pablo M. Romina A. Beatriz M. (2023). Surgical treatment of a patient with a giant choroidal fissure cyst: a case report. Childs Nerv. Syst. 39, 1097–1100. doi: 10.1007/s00381-022-05753-8, 36396771

